# Zinc Electrode Cycling in Deep Eutectic Solvent Electrolytes: An Electrochemical Study

**DOI:** 10.3390/molecules28030957

**Published:** 2023-01-18

**Authors:** Elisa Emanuele, Andrea Li Bassi, Andrea Macrelli, Claudio Mele, Jacopo Strada, Benedetto Bozzini

**Affiliations:** 1 Department of Energy, Politecnico di Milano, Via Lambruschini 4, 20156 Milano, Italy; 2 Department of Innovation Engineering, University of Salento, Via Monteroni, 73100 Lecce, Italy

**Keywords:** Zn, deep eutectic solvent, Zn anode, post-lithium, shape change, water activity

## Abstract

Among post-lithium ion battery technologies, rechargeable chemistries with Zn anodes bear notable technological promise owing to their high theoretical energy density, lower manufacturing cost, availability of raw materials and inherent safety. However, Zn anodes, when employed in aqueous electrolytes, suffer from hydrogen evolution, passivation, and shape changes. Alternative electrolytes can help tackle these issues, preserving the green and safe characteristics of aqueous-based ones. Deep eutectic solvents (DESs) are promising green and low-cost non-aqueous solvents for battery electrolytes. Specifically, the cycling of Zn anodes in DESs is expected to be reversible, chiefly owing to their dendrite-suppression capability. Nevertheless, apart from a few studies on Zn plating, insight into the cathodic–anodic electrochemistry of Zn in DESs is still very limited. In view of developing DES-based battery electrolytes, it is crucial to consider that a potential drawback might be their low ionic conductivity. Water molecules can be added to the eutectic mixtures by up to 40% to increase the diffusion coefficient of the electroactive species and lower the electrolyte viscosity without destroying the eutectic nature. In this study, we address the electrochemistry of Zn in two different hydrated DESs (ChU and ChEG with ~30% H_2_O). Fundamental electrokinetic and electrocrystallization studies based on cyclic voltammetry and chronoamperometry at different cathodic substrates are completed with a galvanostatic cycling test of Zn|Zn symmetric CR2032 coin cells, SEM imaging of electrodes and in situ SERS spectroscopy. This investigation concludes with the proposal of a specific DES/H_2_O/ZnSO_4_-based electrolyte that exhibits optimal functional performance, rationalized on the basis of fundamental electrochemical data, morphology evaluation and modeling of the cycling response.

## 1. Introduction

Deep eutectic solvents (DESs) are green liquid media typically obtained by mixing Lewis or Brønsted acids and bases, of which the reciprocal interactions result in a eutectic with a depression of the melting point significantly below the ideal-mixture value [1]. DESs share many characteristics with ionic liquids (ILs), among which are relevant for battery applications, such as low vapor pressure, good thermal and chemical stability, non-flammability and easy moldability. However, different from ILs, DESs exhibit the key advantage of a simple synthesis based on low-cost, biodegradable and non-toxic raw materials. This advantage can be emphasized further if raw biobased materials are employed (natural deep eutectic solvents, NADES [2,3]). Similar to ILs, DESs are particularly attractive in a wide range of electrochemical technologies, such as (i) electrodeposition of different materials (nanostructures, polymers and coatings [4,5,6]); (ii) battery and supercapacitor electrolytes [7,8,9,10]; (iii) reaction media for synthesis of electrodes and electrocatalysts [2]; (iv) extractants for hydrometallurgy, including battery recycling [11]. The key functional characteristics of DESs for electrochemistry have a wide electrochemical window and an interface that is more structured than that allowed by the classical solvent + supporting electrolyte system ILs [12].

Among battery applications, DESs have been reported as novel electrolytes for rechargeable Zinc-ion batteries (ZIBs) [13,14,15,16,17]. ZIBs typically feature neutral or weakly acidic aqueous electrolytes and exhibit the typical drawbacks of Zn anodes: passivation and dendrite formation. DES-based electrolytes have been reported to enable reversible plating/stripping of Zn and dendrite suppression [2,6].

An advantage of DES solvents with respect to aqueous ones is that the concentration of electroactive species is also critical in view of controlling electrode shape evolution in electroplating. For these reasons, the capability of tuning the coordination chemistry and the metal complex formed between the Lewis acids of the metal and the Lewis base of the DES is key. In this respect, a specific advantage of DESs for metal plating/stripping processes is that they have a high ability to solubilize metal salts, metal oxides and hydroxides and, in correspondence, they impede the formation of insoluble oxides and hydroxides [4,6]. For the case of Zn, EXAFS-based Zn^2+^ ion speciation studies in ChCl:U and ChCl:EG pointed out that, in both DESs, [ZnCl_4_]^2−^ is the dominant species [18,19]. Moreover, the peculiarities of Zn^2+^ coordination were found to result in unusual voltammetric behavior, characterized by a cathodic peak in the cathodic branch of the anodic-going scan. Generally, the mechanistic studies of Zn electrodeposition onto glassy carbon (GC) from DESs [20,21,22,23,24,25] have concluded that the metal is formed by the reduction of an intermediate Zn-containing species. Finally, the reactivity of choline chloride and ethylene glycol can impact coordination in that these species can be dehydrogenated at negative potential forming RO^−^, which can replace one or more chloride ligands in [ZnCl_4_]^2−^ [20,21,23].

As pointed out in the pioneering work described in [3], the physical chemistry of DES solutions can be fine-tuned with the addition of water for electrochemical applications. Water molecules, in even large proportions, can be incorporated into the DES framework to improve the ionic conductivity, increase the diffusion coefficient of specific solutes and lower the viscosity without destroying the eutectic nature of DESs. Specifically, viscosities of DESs are of the order of 1 mPa·s at ambient temperatures [26]: these relatively high viscosity values are mainly due, on the one hand, to the presence of hydrogen bonding and Van der Waals interactions that limit the mobility of ions, and, on the other hand, to the large ion size and small void volume. Generally, high viscosity adversely affects electrochemical processes owing to sluggish mass transport; therefore, the availability of low-viscosity DESs is highly desirable [27].

Neutron diffraction studies [28] reveal that, in the bulk region, choline and urea are bound to chloride atoms in a pure DES, while added water is preferentially associated with the cholinium ion. Thus, water hydrates the pure DES by sequestration around the cholinium cation, but the DES structure is nevertheless maintained up to a water content of ca. 40%. As a result, water participation in the hydrogen bond network strengthens the cohesive interactions that control the liquid structure, increasing the number of coordination layers. When the water content exceeds a critical threshold, the water-in-eutectic systems are disrupted, and an ordinary aqueous solution structure is obtained. The addition of water in DESs has been considered in the fields of electroanalytical sensing [3] and energy storage devices [13,14,16,17].

A convenient way to control the amount of water present in the eutectic mixture is by dissolving hydrated salts. In this work, we shall report the synthesis of hydrated eutectic Zn electrolytes with precise hydration, achieved by mixing a hydrated salt ZnSO_4_·7H_2_O with two of the most common DESs: reline and ethaline, which are stable in an ambient atmosphere.

The link between the chemistry of Zn^2+^ solutions and DESs is considered, and the peculiarities of Zn electrochemistry in these solutions can be rationalized in terms of the DES–electrode interface structure. DES interfaces are inherently more complex than ILs interfaces. Whereas ILs consist of cations and anions only, DESs comprise cations, anions and molecular components that cannot participate in ionic interactions. In the cases considered in this study, DES:EG and DES:U have a concentration of non-ionizable molecules that is twice that of the salt. In a representative investigation on this point, Chen et al. studied a series of DESs at HOPG electrode interfaces as a function of potential, finding multilayer nanostructures with a number of layers and interlayer forces that are lower than typical ILs. Similarly, AFM measurements demonstrated that, at potentials more negative than the point of zero-charge (PZC), the Stern layer is enriched in choline, with charged groups facing the electrode and alcohol groups facing the bulk liquid, while the interfacial layer at strongly negative potentials is rich in hydrogen-bond donors (HBD) [29]. Of course, the liquid structure changes brought about by water addition (mentioned above in this section as relevant for energy storage applications) impact the structure of the DES–electrode interface. Specifically, Hammond et al. [30] reported that the layer thickness in the case of a pure DES in contact with a polycrystalline Pt electrode increases from 0.45 to 0.55 nm in the range of 40–50% of water content.

Thus, the possibility of tuning, on the one hand, the solution chemistry of Zn^2+^-containing electroactive species and, on the other hand, the electrode–electrolyte interfacial structure and chemistry by the combined use of DES and water provides a flexible tool to control Zn plating and stripping processes.

Only a few studies of hydrated DES are currently present in the literature on this topic [31]. Recent reports on DESs used as rechargeable Zn battery electrolytes using various methods/strategies and their key performance descriptors are summarized in Table 1.

Shi et al. reported that DES was based on ZnCl_2_ and acetamide with different amounts of water, and FT-IR, Raman and ^17^O NMR spectroscopy data showed that the addition of small amounts of water below the level yielding direct water–water interaction affects the coordination geometry of Zn^2+^ in a way that impacts Zn plating [13]. In fact, based on the accepted view that Zn electrodeposition is through a combination of desolvation and charge-transfer steps, [ZnCl(acetamide)_2_(H_2_O)]^+^ was shown to undergo successive deamidation and dehydration, yielding [ZnCl(acetamide)]^+^ of which the metal center is finally reduced. Although the most stable species is [ZnCl(acetamide)_3_]^+^, the energy barrier for dehydration is much lower than that for deamidation. For this reason, [ZnCl(acetamide)_2_(H_2_O)]^+^ with lower dissociation energy is the actual electroactive species [17]. The effect of Zn solvation was also observed through its effect on the nucleation overpotential since Zn^2+^ in the hydrated DES exhibits faster desolvation kinetics, which, in turn, leads to a lower nucleation overpotential. Finally, Zhao et al. studied the properties of a “water-in-DES” electrolyte (~30 mol.% H_2_O) based on the urea/LiTFSI/Zn(TFSI)_2_ mixture and found that the preferential interaction between water and metal cations favors interfacial charge transfer [16]. Other examples of hydrated eutectic electrolytes reported in the literature are based on sulfolane (SL)/Zn(ClO_4_)^2^·6H_2_O [33] and Methylsulfonylmethane/zinc perchlorate [34]. However, notwithstanding the important contribution of these seminal papers, the mechanism of Zn electrodeposition in DES has not been conclusively assessed.

The present research is meant to contribute to this field, focusing on Zn electrodeposition from two different, hydrated DESs: ChU and ChEG with ~30% H_2_O. In order to gain control of the impact of the cathode material, we carried out our electrochemical tests by employing GC, Zn, Pt and Au electrodes. Moreover, in order to test the performance of these electrolytes in real-life device conditions, we carried out electrochemical cycling tests in symmetric CR2023 coin cells.

## 2. Results and Discussion

The electrokinetic and electrocrystallization processes controlling Zn electrodeposition were investigated by CV and chronoamperometric measurements. In addition, different working electrode materials were employed in order to deepen the Zn nucleation phenomena.

### 2.1. Cyclic Voltammetry (CV) Experiments

**Figure 1:**
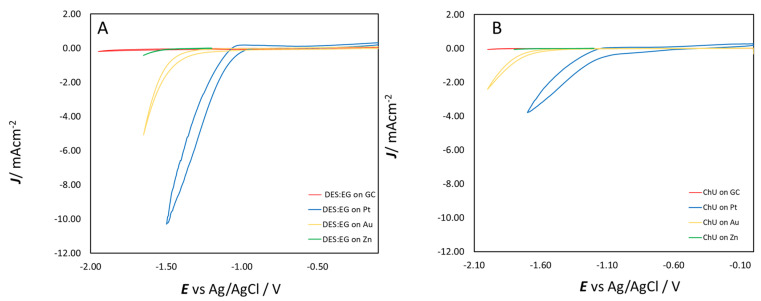
Cyclic voltammograms in the cathodic range for ChEG (Panel (**A**)) and ChU (Panel (**B**)) with GC, Pt, Au and Zn electrodes.

Prior to working with Zn^2+^ in solution, the electrochemical windows of the two DESs were explored with CV at different working electrodes: Zn, GC, Au and Pt (see Section 3.3.1). These hydrated eutectic electrolytes exhibit conveniently wide potential windows of ca. 3.0 V for electrochemical experiments with the Zn–Zn^2+^ couple out of equilibrium. The cathodic potential limits reported in Figure 1 for ChEG (Panel A) and ChU (Panel B) can be attributed to the reduction of choline ions (Ch^+^), ethylene glycol or water. The electrochemical decomposition of the quaternary ammonium salts can lead to the formation of the choline radical that, in turn, can decompose to trimethyl amine and ethanol radical or to dimethylaminoethanol and a methyl radical [34]. The formation of a methyl radical can then form different side products, such as CH_3_Cl or CH_4_. Water reduction, instead, increases the concentration of OH^−^ at the double layer, and with a formation of trimethylamine, the Hoffman elimination is likely to occur, too. Pt is known for its excellent electrocatalytic properties for the hydrogen evolution reaction (HER), and since water reduction is one of the possible reactions taking place at high cathodic potentials in the investigated range, the highest increase in background current is shown at the platinum electrode. The ChEG electrolyte yields higher CDs than ChU, owing to the different viscosities of the two media; in fact, ethylene glycol is a weaker hydrogen-bond donor (HBD) than urea.

**Figure 2:**
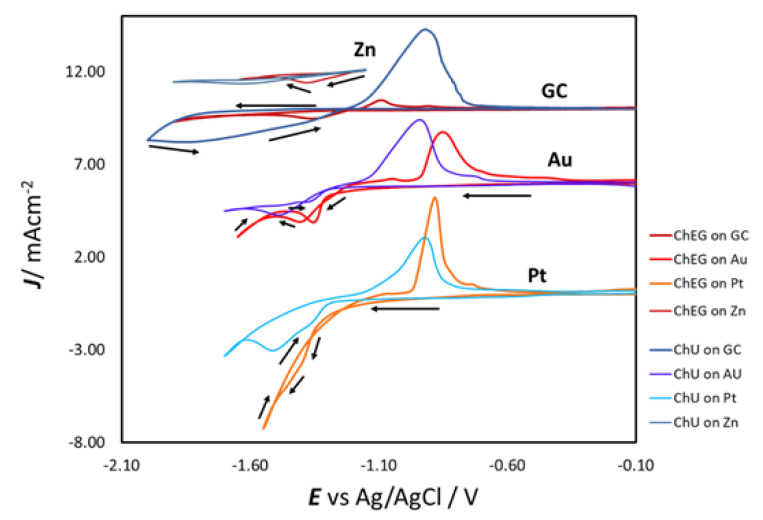
Cyclic voltammograms of 0.3 M ZnSO_4_ in ChU (blue shades) and 0.3 M ZnSO_4_ in ChEG (red shades) with GC, Au, Pt and Zn electrodes. The arrows indicate the direction of the cycle.

CVs of ChU and ChEG with 0.3 M ZnSO_4_ for all working electrodes used are reported in Figure 2. The experiments have been repeated three times and proved to be perfectly reproducible (only one curve out of three is shown for clarity). The voltammetric response varied depending on the substrate on which zinc was deposited. The electrochemical behavior on Zn exhibits a CV pattern, typical of electrodeposition on an identical cathode with an onset potential of −1.22 V for both electrolytes. The cathodic peak is centered at −1.62 V for ChU and −1.37 V for ChEG. Our results contrast the study results of [20] but agree with those of [25]. A possible explanation could be the use of different Zn salts; Indeed, for the case of ILs, reference [35] pointed out that the counterion of the Zn^2+^ salt drastically affects the zinc electrochemistry on a GC substrate. Zn deposition on Pt shows different CV patterns in the two electrolytes. If ChEG is used, a CD crossover is present with only one cathodic peak at −1.40 V. Instead, in ChU, one shoulder is found at −1.37 V and a cathodic peak at −1.51 V, while the CD crossover is not present. In both cases, the cathodic peak is followed by an anodic one, centered at −0.90 and −0.81 V for ChU and ChEG, respectively. This difference will be explained below.

The CVs on GC for both electrolytes show that the cathodic peaks at −1.75 V (ChU) and −1.40 V (ChEG) are observed only after reversing the scan. The reduction peak is followed by an oxidation one with a current peak located at −1.00 V. This unusual voltammetric behavior, where the reduction peak appears on reverse anodic scans, has also been reported for other DESs and ILs [20,21,23,24,36]. In particular, Zn deposition from 1-ethyl-3-methylimidazolium dicyanamide [emim][dca] IL showed a cathodic peak during the reverse scan [36]. This behavior is due to the fact that the compact layer is depleted of Zn^2+^ species in the double-layer charging region and is thus excluded from electroactivity until a potential is reached, at which the reaction of the compact layer species enables access of Zn^2+^ and can thus undergo reduction. In parallel, Pereira et al. [23] reported the same voltammetric pattern on stainless steel and justified this behavior with the potential-dependent formation of a choline layer and Zn intermediates: a compact choline layer forms at equilibrium and is desorbed at some cathodic potential beyond which a reducible zinc species can reach the electrode surface. The same authors also pointed out that additives, such as dimethylacetamide, considerably change the CV shape. The reason for this phenomenon could be that the additive interferes with choline adsorption, impeding the formation of the choline compact layer and, therefore, enabling a Zn^2+^ reduction at less cathodic potentials. Since the reduction of water in these conditions is one of the possible reactions taking place at high cathodic potentials, the electrode surface could exhibit a partial coverage with adsorbed H and choline. The Pt electrode, exhibiting the highest metal–hydrogen bond strength, is expected to emphasize this phenomenon, and indeed, our results showed that—at the Pt electrode—the reduction peak is already present during the forward cathodic scan (Figure 2).

**Figure 3:**
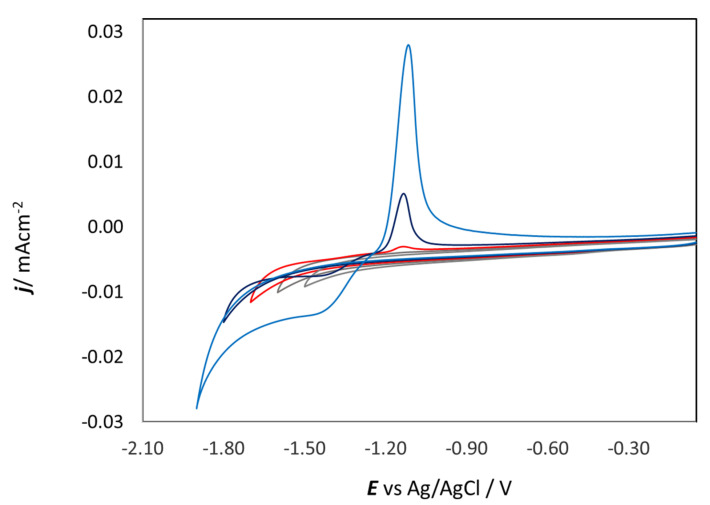
Cyclic voltammograms of 0.3 M ZnSO_4_ in ChU with GC electrode at 25 °C and 10 mV s^−1^ and different cathodic vertex potentials.

**Figure 4:**
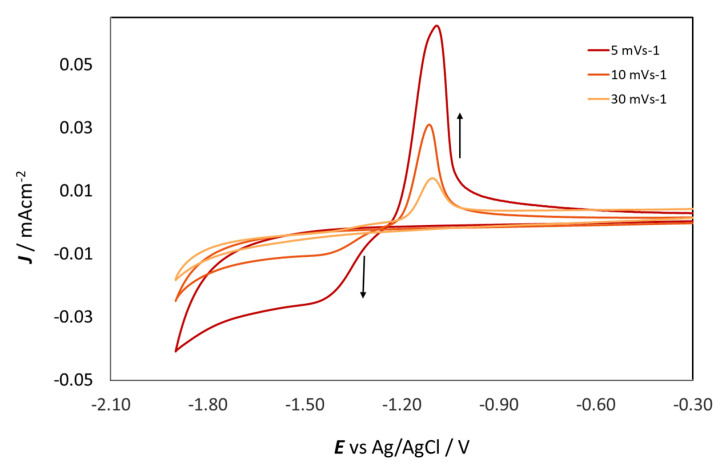
Cyclic voltammograms of 0.3 M ZnSO4 in ChU with GC electrode at 25 °C at different scan rates: 5 (Red), 10 (orange) and 30 mV s^−1^ (yellow). The arrows indicate the current density increase.

When ZnSO_4_ is dissolved in DESs, the [ZnCl_4_]^2−^ species is formed and stabilized by choline [18,19]. Although tetrachlorozincate is the dominant species, it does not seem to be the complex from which Zn is deposited; indeed, [ZnCl_4_]^2−^ has a very negative reduction potential. Vieira et al. [20,21] suggested the formation of an intermediate electroactive species triggered by the cathodic reaction of the electrolyte. Figure 3 shows CVs in which the cathodic limit was gradually increased from −1.5 to −1.9 V, and one can notice that the CD at the anodic peak increases with the lower vertex potential. At the same time, the reduction peak at −1.5 V is only visible at lower scan rates, which is anticorrelated with the CD (Figure 4). In any case, in Figure 3 and Figure 4, no reduction peak could be detected in the investigated ranges of cathodic vertex potential and scan rate. From these results, it can be inferred that the formation of the electroactive intermediate resulting from the replacement of one or more chloride ligands in [ZnCl_4_]^2−^ with a cathodic reduction product of EG and/or Ch should be a reaction with slow kinetics. Chronoamperometric measurements on GC, which will be discussed in Section 2.3.1, showed that Zn deposition is already present at −1.35 V. Therefore, the formation of the electroactive intermediate also seems to show a notable time dependence.

**Figure 5:**
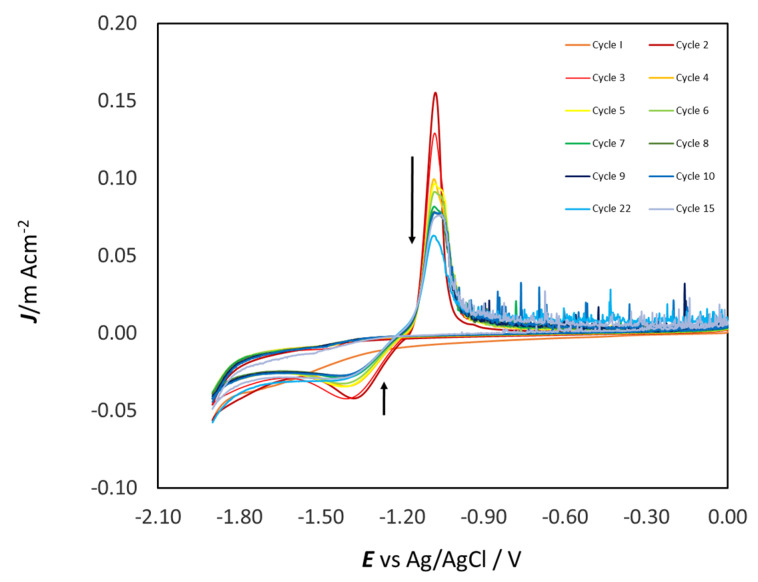
Multiple-cycle voltammograms in 0.3 M ZnSO_4_ in ChU with GC electrode (first: red line, last: blue line) at 25 °C and 10 mV s^−1^.

Multiple CVs at a GC WE (Figure 5) progressively develop electrochemical noise in the anodic region, which can be attributed to the rupture of ZnO film produced in the anodic process. A similar dynamic process was observed in an alkaline aqueous solution [37]. In correspondence, the peak CD decreases with the number of cycles (from red to blue), and the cathodic peak is present in the reverse scans of all cycles.

On Au, CVs show a cathodic peak in the cathodic forward sweep as well as in the anodic reverse one. This behavior has also been reported in previous studies [20]. Apparently, two different effects are present at the same time in this material. Since, as stated above, the formation of the more reducible Zn species correlates with DES degradation that can be triggered by proton reduction, differences in electrocatalytic behavior for HER among the cathodic substrates considered can explain the different encountered CV patterns [38]. Moreover, the cathodic peak visible with an Au electrode in the anodic-going scan following the attainment of the cathodic vertex potential can be due to the formation of an adsorbed layer. In particular, a specific interaction can develop between the lone pairs of gold and nitrogen, favoring the formation of an interfacial organic layer. Scanning further into the anodic range can lead to the desorption of this organic layer and electrodeposition from the newly-formed electroactive Zn(II) intermediate, which could access the double layer in these conditions.

In addition to the above-mentioned differences in CV patterns between the DESs investigated, one can notice a potential systematic shift: the cathodic peak is always found at more positive potentials when ChEG is used. This shift can be attributed to the lower viscosity and lower electrochemical stability of this electrolyte with respect to ChU.

### 2.2. SERS Analysis of Au–DES Interface

**Figure 6:**
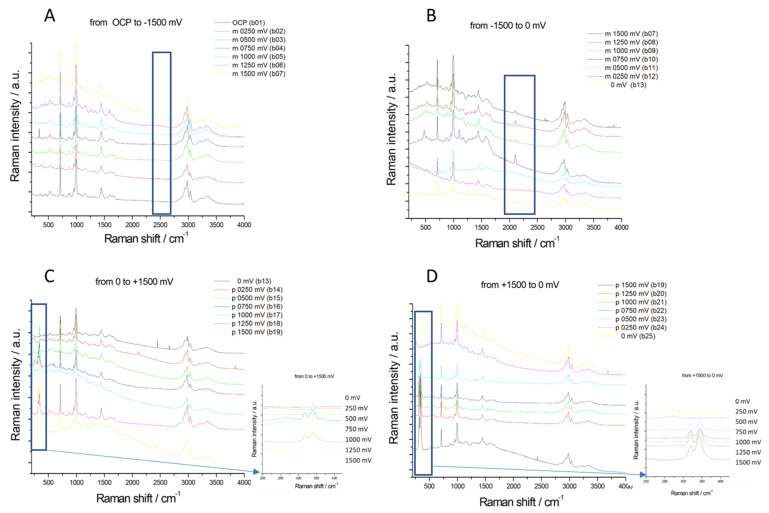
Potential-dependent in situ SERS spectra were measured at an Au electrode in contact with the DESU electrolyte. Panels (**A**–**D**) report the spectra recorded in the whole measured range. Blue rectangles highlight diagnostic spectral ranges.

**Figure 7:**
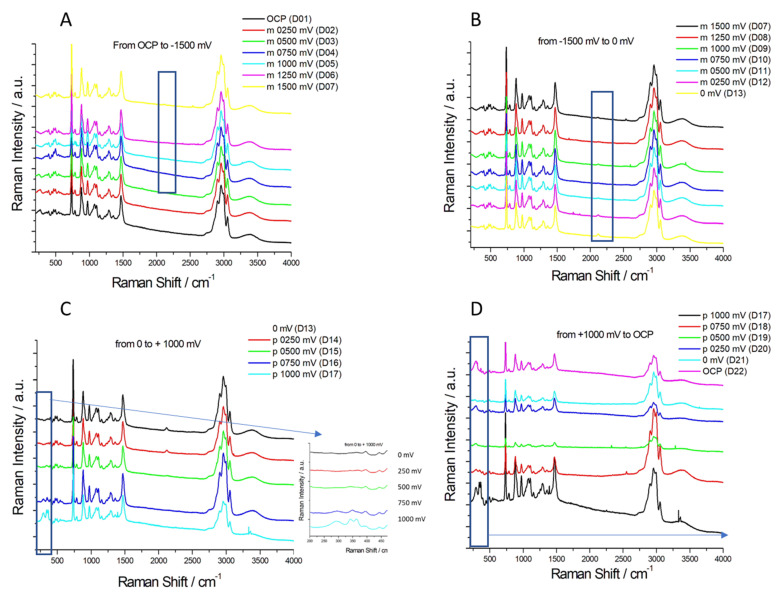
Potential-dependent in situ SERS spectra measured at an Au electrode in contact with the DESEG electrolyte. Panels (**A**–**D**) report the spectra recorded in the whole measured range. Blue rectangles highlight diagnostic spectral ranges.

In order to clarify the role of surface reactivity and orientation of the investigated DESs, Surface Enhanced Raman Spectroscopy (SERS) was employed: the SERS-active Au electrode was selected for study [39,40]. Figure 6 and Figure 7 report potential-dependent SERS spectra, measured in situ in the potential range investigated by CV in Section 2.1 with the two DES-based electrolytes studied in this work. Vibrational bands typical of C-N single bond, symmetric (712 cm^−1^, 864 cm^−1^) and asymmetric (952 cm^−1^), C-H stretching (2922 cm^−1^) can be assigned to the hydrogen-bond acceptor (HBA) (Ch^+^), while C-N symmetric (1010 cm^−1^) and asymmetric (1450 cm^−1^) stretching, NH_2_ asymmetric and symmetric deformation (1589 cm^−1^, 1647 cm^−1^) and C=O stretching (1683 cm^−1^) can be assigned to the HBD (urea). Ethylene glycol SERS spectra are instead characterized by C-O stretching in the region between 1000 and 1100 cm^−1^, a very intense Raman band at 2958 and 2997 cm^−1^ corresponding to the stretching mode of CH_2_ groups and a broader peak corresponding to O-H stretching. The potential-dependent SERS spectral features are similar for both electrolytes. Upon polarization in the high cathodic range at −1500 mV, a new peak at 2132 cm^−1^ can be observed, which is characteristic of ν(C≡N) of adsorbed CN^−^ ([41] and references therein contained); this anion results from the degradation of quaternary ammonium salts. As discussed in Section 2.1, different reaction mechanisms can lead to the formation of CN^−^. Hoffman’s elimination of choline cations is likely to occur at high cathodic overvoltage conditions where the concentration of OH^−^ is higher because of HER, leading to trimethylamine formation. The thus-formed trimethylamine can then be deprotonated by either OH^−^ or H^•^ to form the CN^−^ group. In the range from −1500 to 0 mV, there is a slight blue Stark shift of the vibrational frequency of the nitrile stretching mode that is characteristic of adsorbed CN^−^ and has been widely investigated in aqueous solutions ([42] and references therein) but also evidenced in ILs electrolytes [39,40]. The CN^−^ band is also present when the potential is switched anodically at 0 and +250 mV. In fact, once CN^−^ has been formed at −1500 mV, this anion remains adsorbed until anodic desorption settles. Upon anodic polarization at potentials higher than 750 mV, new bands that form at 270, 319 and 340 cm^−1^ (Figure 6 (Panels C, D) and Figure 7 (Panels C, D)) corresponding to the Au-Cl stretching and bending resulting from the partial oxidation of the Au surface. This result is in line with the peculiarities of the CV pattern reported in Figure 2.

### 2.3. Nucleation and Growth of Zn

#### 2.3.1. Chronoamperometry (CA)

**Figure 8:**
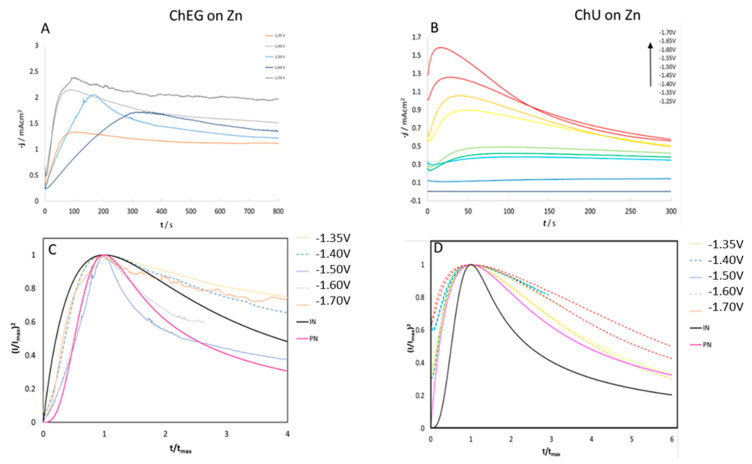
(**A**,**B**): Chronoamperograms for 0.3 M ZnSO_4_ in ChEG (**A**) and ChU (**B**) using a Zn electrode at 25 °C. (**C**,**D**): Normalized experimental curves of Panels (**A**,**B**), plotted together with theoretical curves for progressive (solid pink line) and instantaneous nucleation (solid black line) according to the model of [32,33].

**Figure 9:**
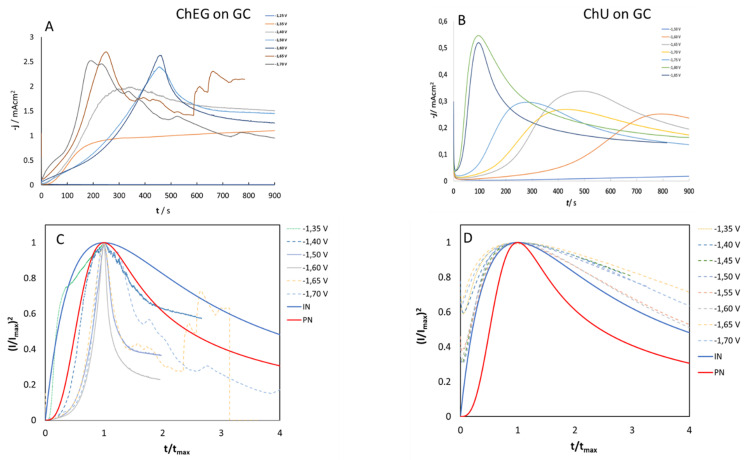
(**A**,**B**): Chronoamperograms for 0.3 M ZnSO_4_ in ChEG (**A**) and ChU (**B**) using a GC electrode at 25 °C. (**C**,**D**): Normalized experimental curves of Panels (**A**,**B**), plotted together with theoretical curves for progressive (solid pink line) and instantaneous nucleation (solid black line) according to the model of [32,33].

CA experiments were performed in ChU and ChEG electrolytes to study the nucleation and growth mechanisms of Zn electrodeposition on Zn and GC substrates. In Figure 8A,B and Figure 9A,B, we report CD-time transients obtained by stepping the potential from an initial value of −1.25 V, where reduction reactions cannot take place, to values that are sufficiently negative to initiate nucleation and growth of Zn crystallites. It is worth noting that the CD surge observed at the start of the experiment is due to the charging of the electrochemical double layer. Nucleation and growth of electrodeposited Zn onto polycrystalline Zn and GC electrodes were analyzed according to the Scharifker–Hills (SH) three-dimensional (3D) nucleation models [43,44] and considering two limiting cases: instantaneous nucleation (IN) and progressive nucleation (PN). In the former case, all nuclei are formed immediately upon application of the potential step; in the latter case, the formation of nuclei occurs over a time span (i.e., the number of nuclei increases during the deposition process).

According to the analysis of [37,38], who combines geometrical assumptions with diffusive mass-transport theory, the analytical forms of the transients for IN or PN nucleation are given by Equations (1) and (2):

(1) $$\mathrm{IN}:{\left(I/I_{\max}\right)}^{2}=1.9542\;{\left(t/t_{\max}\right)}^{-1}\;{\left[1-\exp\left(-1.2564\;t/t_{\max}\;\right)\right]}^{2}$$

(2) $$\mathrm{PN}:{\left(I/I_{\max}\right)}^{2}=1.2254\;{\left(t/t_{\max}\right)}^{-1}\;{\left\{1-\exp\left[-2.3367{\left(\;t/t_{\max}\right)}^{2}\;\right]\right\}}^{2}$$

In order to discriminate between these two nucleation processes, an effective semi-quantitative approach is that of comparing the normalized experimental current transients with the theoretical ones (Figure 8C,D and Figure 9C,D). Overall, the experimental data for the ChU electrolyte are approximated well by the 3D instantaneous nucleation model for the potential range studied on both Zn and GC electrodes, whereas the whole set of obtained nucleation transients with the ethylene-glycol-based DES cannot be followed with either the IN or the PN nucleation model (a similar case is not uncommon, as documented in [18]). Zn deposition from the ChEG electrolyte onto the Zn electrode seems, in fact, to follow two different nucleation mechanisms depending on the potential applied: CD transients at −1.3 and −1.4 V exhibit the IN features, while potentials more cathodic than −1.5 V, the PN mechanism seem to prevail.

As far as the assignment of a nucleation mechanism is concerned, the literature on Zn deposition on extraneous cathodes is not unanimous. Smith et al. [45] have studied the deposition of zinc onto a gold electrode from ChEG, and the experimental data could be accurately fitted with the PN model, while Vieira et al. [20] found the IN mechanism with stainless steel cathodes and the PN one with gold. On this background of the literature, it is clear that a complete understanding of the nucleation mechanism of Zn on different cathodes from DES media, enabling discussion in a general mechanistic framework, is still missing.

It is worth noting that for t/t_max_ > 4, when 3D growth sets in, the transient shape slightly deviates from that predicted by the electrochemical nucleation model based on Cottrell-type diffusion control. Assuming that diffusive mass transport still holds, these deviations indicate that—in the potential range of interest—Zn growth on polycrystalline Zn exhibits an additional activation contribution. Similar deviations from the IN trend are known in Zn nucleation studies [20].

Regarding the nucleation model constants, the diffusion coefficient D (cm^2^ s^−1^) can be estimated by measuring the j_max_ and t_max_ values from Figure 8 and Figure 9 and by substituting them into Equation (3) [43]:

(3) $${\left(I_{\max}\right)}^{2}t_{\max}=0.1629{\left(\mathrm{zFc}\right)}^{2}D\;$$

The average value of the diffusion coefficient in ChU is 5.2 × 10^−8^ cm^2^ s^−1^, which is higher than the one reported in the work of Yang et al. [46]. This result is consistent with the fact that our DES was prepared in the laboratory ambient when adding ZnSO_4_·7H_2_O without drying the salt powder; the water content of the DES can thus increase the diffusion coefficient of Zn^2+^.

#### 2.3.2. SEM Analysis of Zn Electrodeposits

**Figure 10:**
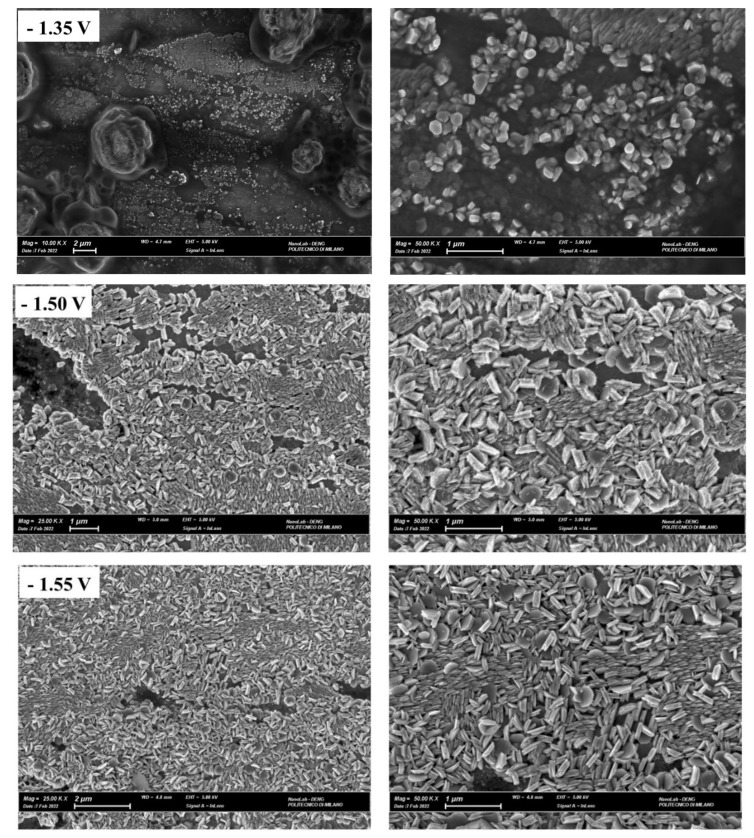

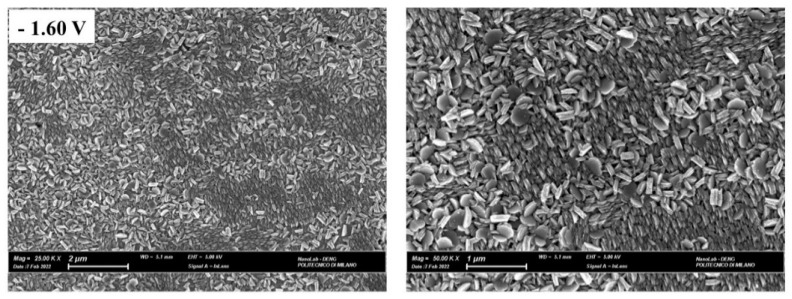
SEM images of Zn electrodeposits were potentiostatically obtained on polycrystalline Zn at E = −1.35, −1.50, −1.55 and −1.60 V from 0.3 M ZnSO_4_ in DES ChU. Electrodeposition time: 900 s.

**Figure 11:**
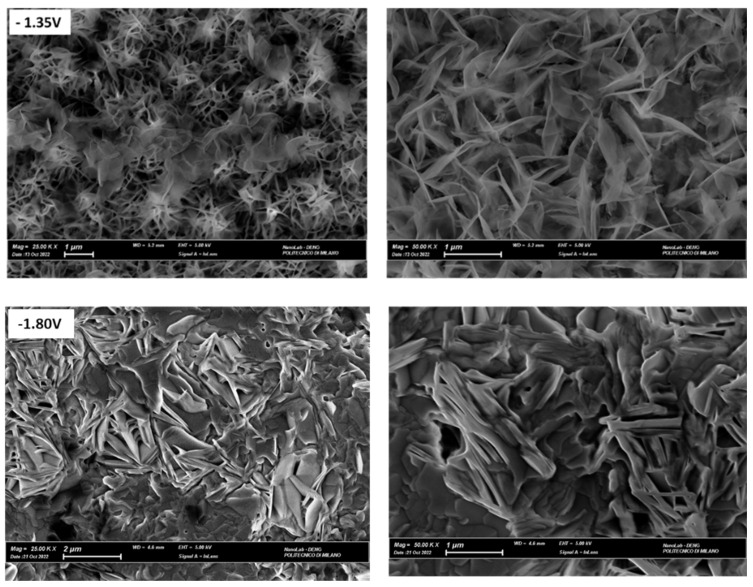
SEM images of Zn deposits were potentiostatically obtained on polycrystalline Zn at E = −1.35 and −1.80 V from 0.3 M ZnSO_4_ in DES ChEG. Electrodeposition time: 900 s.

The morphology of Zn layers grown onto polycrystalline Zn from the two electrolytes considered at different potentials has been characterized by SEM. The images recorded show that the zinc layer deposited from ChU (Figure 10) is formed by hexagonal crystallites. The main impact of the four potentials considered is the surface coverage with Zn crystallites. SEM observations also confirm the nucleation mechanism. Indeed, in the IN cases recognized in Section 3.3.1, since all the nuclei are of the same age and grow at the same rate, crystallites of the same dimensions were found (Figure 10). The morphology of the Zn films deposited from ChEG (Figure 11) at the low cathodic overvoltage of −1.35 V exhibits well-defined individual crystallites of the desert rose type, coherent with an IN mechanism. Instead, at higher cathodic potentials, a compact Zn layer forms, consistent with the PN model.

### 2.4. Galvanostatic Charge-Discharge Cycling (GCD) in Symmetric 2032 Coin Cells

#### 2.4.1. Electrochemical Experiments

**Figure 12:**
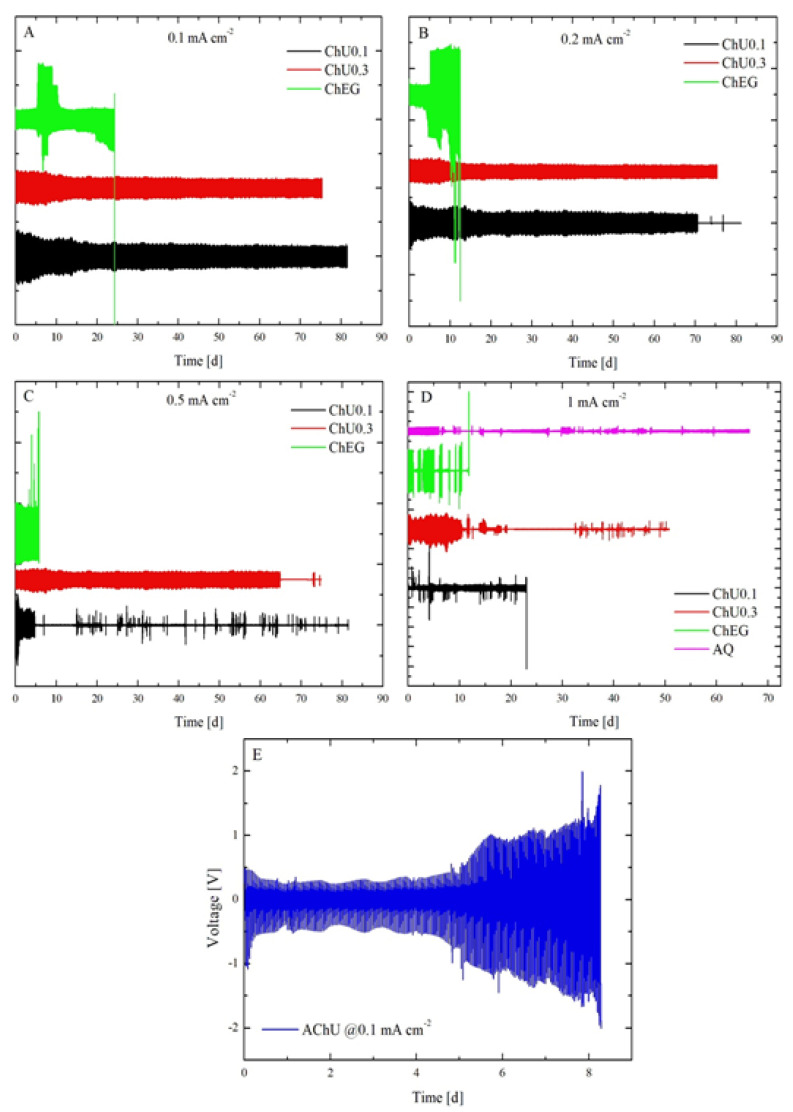
Galvanostatic charge-discharge cycling experiments carried out with Zn symmetric CR2032 coin cells with ChU0.1 (black plot), ChU0.3 (red plot), ChEG (green plot), and ChU electrolytes at 0.1 (Panel (**A**)), 0.2 (Panel (**B**)), 0.5 (Panel (**C**)), 1 (Panel (**D**)) and 0.1 mA cm^−2^ (Panel **E**), respectively.

**Figure 13:**
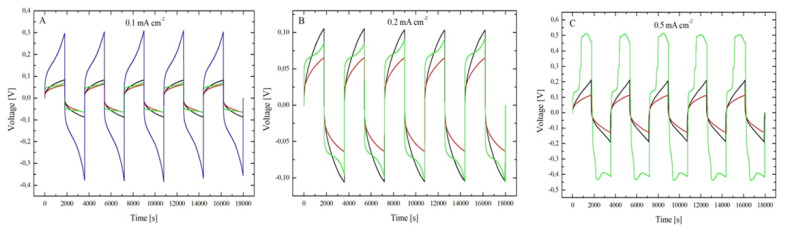
(**A**–**C**) Chronopotentiometric transient shapes are highlighted from the datasets in Figure 12.

Figure 12 and Figure 13 show the corpus of experimental data obtained in the cycling experiments detailed in Section 3.2 and Section 3.3.3.

Eutectic electrolytes based on hydrated electrolytes are found to yield the longest-living cells. With ChU0.3 (red plots in Figure 12), all cells responding with a voltage excursion of ca. ± 70 mV cycled at 0.1 and 0.2 mA cm^−2^ (Figure 12 Panels A and B, respectively) and were still running after 75 days. The chronopotentiometric transients remained stable from the beginning to the end of the test. Again, with ChU0.3, cells cycled at 0.5 and 1 mA cm^−2^, giving rise to voltage responses of ca. ± 120 and ± 300 mV (Figure 12, red plot, Panels C and D, respectively), and were terminated by a short circuit after 58 ± 7 and 7 ± 4 days, respectively (time between the start and the first short circuit). Cell termination occurred from a short circuit within two charge-discharge periods (2 h), exhibiting the irregular voltage behavior highlighted in Figure 12, Panels C and D.

Instead, the coin cells containing ChU0.1 (a urea-eutectic electrolyte with 0.1 M ZnSO_4_ and, therefore, lower hydration level; black plots in Figure 12) show an extension of the period in which the voltage response of the cell has regular behavior. In fact, a short circuit occurs after 33 ± 29 and 3 ± 2 days of cycling at 0.2 and 0.5 mA cm^−2^, respectively. Similar to what we observed for ChU0.3 and ChU0.1, the shape of the transient remains regular and stable (Figure 12 Panels A–D, black plot) until a short circuit occurs after a couple of irregular cycles (see plot in Figure 12). At 1 mA cm^−2^, the cycles are characterized by square transients of ±50 mV from the beginning of the test (Figure 12 Panel D), suggesting ohmic control, possibly due to the presence of a passivation layer, which is, however, fully recovered after a few transients. The fact that the chronopotentiometric transients remain regular until cell failure for the systems analyzed implies a reversible plating/stripping process, as suggested by [47]; If compared with similar experiments in an aqueous solution [47], this indicates that urea-based systems are indeed very promising in view of the stable cycling of Zn anodes. Zn cycling stability is further witnessed by the fact that none of the investigated cells fails by passivation, which is the dominating failure mode for Zn aqueous cells [46,47]. The reason for Zn’s cycling behavior lies in the fact that, in the DESs considered in this study, a small percentage of water has very low activity because of its coordination function when water enters the lattice through hydrogen bonds [2,48]. Indeed, due to its bipolar nature, water molecules can act either as HBD or HBA. Specifically, when water is present in a small amount (<5 w/o or <30 mol.%), it is absorbed in the molecular matrix of DES by forming H-bonds with the ions and HBDs. Zhekenov et al. [48] observed similar behavior for different DESs, considering the number of hydrogen bonds forming in water-containing pairs: HBD–water, choline ion–water and chloride ion–water. Of course, all these interactions increase with increasing water content, and the HBD–water pair demonstrates the most significant change with the formation of a high number of hydrogen bonds as water is added into the system; this is also reflected in the high solubility of the components in water. Furthermore, the other component pairs (i.e., HBD–choline ion, HBD–chloride ion and HBD–HBD) have a descending trend in molecular interactions with the addition of water for all DESs. A decrease in these interactions reflects the preference of HBD to form hydrogen bonds with water, owing to its high polarity, rather than with other species present in the system. An alternation is observed of potential maxima and minima during the entire test period when looking more closely into the long-term cycling response for all CDs. According to [49], this indicates a stable plating/stripping process that corresponds to anode durability. Moreover, we observed that the sequence of potential maxima and minima exhibits a slight seasonal trend of ca. 40 mV, with a period of 1 day linked to the cyclic variation of the ambient temperature. This has been demonstrated with a dedicated experiment in a climatic chamber at a fixed temperature (25 °C), which resulted in the suppression of the seasonality.

Regarding ChEG (green plots in Figure 12), the typical cell-termination mode is by passivation; this can be clearly noticed for cycling tests at 0.1, 0.2 and 0.5 mA cm^−2^, yielding lifetimes of 32 ± 7, 21 ± 15 and 4 ± 1 day(s), respectively. Instead, at 1 mA cm^−2^, a short circuit is reached at 16 ± 12 h. The short circuit is of the temporary type due to dissolution at OCP of the metal filaments connecting the two electrodes, and after a sequence of a few recovered short-circuit events, the cell finally failed by passivation. Quite consistently, all three replicates exhibit an alternation between a short circuit and chronopotentiometric transient (after the first regular ones), which lasted about 22 ± 9 cycles. Finally, after 13 ± 3 days the cells reach the cut-off voltage. For 0.1 and 0.2 mA cm^−2^, the transient amplitude goes from ca. ±70 mV up to ca. ±400 mV; instead, for 0.5 and 1 mA cm^−2^, the potential excursion is ca. ±500 mV. Moreover, the cells with EG-based electrolytes sometimes undergo sudden potential excursions—in which both the transient amplitude and shape change and then relax after relatively long periods of ca. 9 days—that denote more unstable behavior of Zn with respect to the smooth potential time series observed with U-based electrolytes.

In the borderline AChU case (blue plots in Figure 12), a higher internal resistance prevents the cells from cycling unless low currents are applied; at 0.1 mA cm^−2^, a gradual overpotential growth is observed, leading to the attainment of the cut-off voltage after 9 ± 2 days. Instead, at 0.2, 0.5 and 1 mA cm^−2^, the cells reach 2 V right at the beginning of the experiment, denoting high internal resistance. These measurements demonstrate the effect of water within the DES. Indeed, according to [50], the controlled addition of water inside the DES tunes the conductivity of the electrolyte. Comparing Figure 12A,E, it is clear how the conductivity, and thus the ohmic resistance of the electrolyte, plays a fundamental role in cell operation.

If, on the other hand, the anodic–cathodic switching regime is taken into account (Figure 13), it is clearly visible that DES systems have very similar and stable shapes of the potential transients. In particular, a good symmetry of the shape of the anodic and cathodic transients is noted for U-systems. This behavior suggests good reversibility of the plating/stripping processes, also visible at 1 mA cm^−2^ for ChU0.3. For ChEG, the chronopotentiometric transients remain regular and symmetric only at low CDs and for a limited period of time; the starting 54 ± 47 cycles at 0.1 and 0.2 mA cm^−2^ are represented in Figure 13 in green. Instead, at 0.5 mA cm^−2^, the shapes of the cathodic and anodic branches of the transients are no longer symmetric, suggesting that—at high CDs—EG gives rise to a less stable behavior than U. The same observations also hold for the tests carried out at 1 mA cm^−2^, with the difference that after the first short circuit, the cells resume a regular cycling behavior but with a different transient shape, changing from a simplified arc-shape to a double-peak one. Comparing ChU0.3 instead with AChU, the second exhibits a similar transient shape but with faster growth due to the presence of more pronounced mass transport effects.

Chronopotentiometric transients were analyzed using the approach and model reported by Bozzini et al. [49]. For parameter-estimation purposes, the time dependence of the solutions can be separated into a slow regime, corresponding, on the one hand, to dendrite or mossy metal growth and, on the other hand, to passivation and a fast regime controlled by cathodic and anodic electrokinetics, mass-transport and nucleation. The fast regime involves the timescale of a single galvanostatic interval (a half period of the galvanostatic square wave) and controls the shape of each individual chronopotentiometric transient. According to the shape of the galvanostatic switching transients, it is possible to classify the cell response dynamics according to representative ranges of model parameters accounting for mass-transport (diffusivity D) and electrocrystallization (maximum radius of nuclei, r_max_, and rate of hemispherical bulges formation, k_0,hs_). The long-term regime describing the evolution of the time series over several GCD cycles is quantified with the metal outgrowth rate, F_C_, and the passivation rate, k_pass_.

Based on this model, most of the transients reported in Figure 13 can be traced back to an arc-shape transient, and from the comparison between our results and what is reported in [49], it can be concluded that the transient shape is dominated by the low diffusion coefficient of Zn^2+^ in U-based DESs. This result is in accordance with the fact that DESs have higher viscosity and lower conductivity compared with other ionic liquids [6,51] or water [49]. Subsequently, it is observed that the diffusion coefficient decreases from ChU to ChEG, as expected from the fact that ChEG has a higher conductivity than ChU. Based on the shape of the transients reported in Figure 12 and Figure 13, the other parameters of the key model were classified as detailed in Table 2.

As far as the long-term GCD cycling behavior is concerned, the qualitative observations on outgrowth and passivation propensity found in the time series of Figure 12 can be quantitatively framed with the model of [48] and with the parameter assignment of Table 3.

#### 2.4.2. SEM Analysis of Zn Electrode

**Figure 14:**
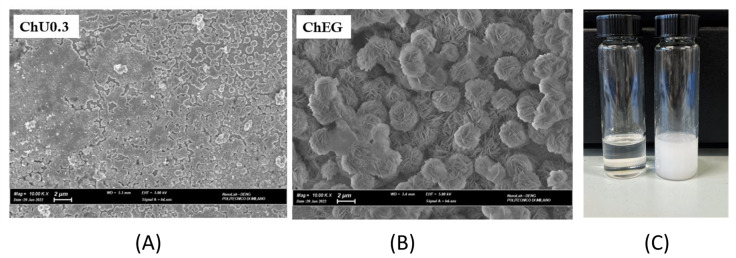
SEM images of Zn anode extracted from the cells with ChU0.3 and ChEG electrolytes cycled at 1 mA cm^−2^ in the experiments reported in Figure 12 (Panels (**A**,**B**)) and dissolution of 10 mg ZnO in 10 mL of ChU0.3 (left side) and ChEG (right side) (Panel (**C**)).

Post-mortem analysis of the structure and morphology of the Zn electrodes cycled in the experiments described in Section 2.4.1 and extracted from the coin cells were investigated by SEM. Figure 14 shows the typical morphology of the central area of the dismounted Zn anode electrode cycled at 1 mA cm^−2^ in two different electrolytes. The electrode morphologies obtained in the two electrolytes are deeply different. Zn cycled in the ChU0.3 liquid exhibits homogeneously distributed globular grains. Instead, in ChEG, the Zn surface is covered by flat patches of poorly conducting electronic material. In the latter case, the overall morphology seems to be controlled by the growth of a ZnO layer during the discharge’s half cycle onto electrodeposited Zn, produced during the charge interval. This can be straightforwardly explained by the fact that DESs have a high solubility not only for metal salts but also for oxides and hydroxides. This gives an advantage over aqueous and organic electrolytes with respect to anode passivation issues; however, ZnO solubility in ChU0.3 is much higher than in ChEG. A simple experiment was conducted to prove that the solubility of ZnO can be affected by the two different electrolytes, where 10 mg of ZnO was dissolved in 10 mL of electrolyte and left at 70 °C overnight (Figure 14 Panel C). After the stirring and heating process, the ChU0.3 DES appears transparent, while ChEG looks milky white.

As far as Zn grain size is concerned, this increases in the order of ChEG < ChU0.3. The grain size and its relationship with nucleation mode can be correlated to the viscosity of the system used, and it—according to the theory of [43,44]—has a bearing on the nuclei’s number density. ChU0.3 exhibits the highest viscosity among the investigated DESs, which could explain why the largest Zn grain sizes have been observed with this electrolyte. Moreover, the nucleation overpotential can be estimated by analysis of the rising front of cell voltage after the subtraction of ohmic contribution. In alkaline aqueous electrolytes, the nucleation overpotential decreases from 190 mV in DESU0.3 and 180 mV in DESEG electrolytes to 70 mV. Although the dissolution of metal oxide in DES seems to be a common practice in metal electroplating, only a few studies concerning the dissolution mechanisms and dissolved species are available. In particular, [51] shows that the solubility of ZnO increases in the urea > ethylene glycol order, coherent with our observation of Figure 14 Panel C. This result fits well with the literature’s data on ZnO solubility in ChU and ChEG [52], even if a clear explanation of the influence of HBD in the solvation mechanism was not reported. Similar to ILs, the availability of coordinating ligands appears to be important for the dissolution of metal oxides. The high solubility of transition-metal oxides is, in some cases, due to their ability to complex with urea. When ZnO dissolves in the deep eutectic solvent, the oxygen remains attached to the metal center, and urea acts as a ligand forming [ZnClO(urea)]^−^. In addition, [53] reports that urea is a stronger HBD than ethylene glycol, and its higher electronegativity can be used to explain the dissolution mechanism. Indeed, Zn is a “late” and “hard” metal (i.e., a relatively electronegative metal, sitting on the right-hand side of the periodic table that tends to retain its valence electrons), which tends to form more stable complexes with a “hard” ligand (i.e., ligands that are small, difficult to polarize and predominantly form ionic bonds). These species bind better to “hard” cations, such as H^+^ and Zn^2+^, which are also small and difficult to polarize. We can thus conclude that the coordination with a stronger ligand, such as urea, results in better passivation suppression. The milky-white appearance of the ChEG + ZnO can thus be attributed to the formation of a stable colloidal suspension rather than a complete dissolution process. Ethylene glycol can act more like a suspending agent rather than as a complex former for Zn^2+^. Indeed, some of the literature reports the use of polyol for the preparation of metal-oxides nanoparticles, yielding a stable colloidal suspension [54]. Given the above, the cell cycling behavior can be explained on the basis of a higher solubility of ZnO in ChU, resulting in the fact that cycling tests performed with U-series electrolytes exhibit more regular cathodic–anodic potential transients and failures by short circuits. Instead, the cycling of cells with EG-series electrolytes is characterized by more irregular potential transients (especially at high CDs) and failure by passivation.

## 3. Materials and Methods

### 3.1. Preparation of Deep Eutectic Solvents, Electrolytes and Electrodes

Three different DES electrolytes were used: hydrated 0.3 M ZnSO_4_ in reline (abbreviated ChU0.3), anhydrous 0.3 M ZnSO_4_ in reline (abbreviated AChU), 0.1 M ZnSO_4_ (abbreviated ChU0.1), and 0.3 M ZnSO_4_ in ethaline (abbreviated ChEG). Reline was first prepared by combining urea (CO(NH_2_)_2_, ACS (New York, NY, USA) reagent, 99%) and choline chloride (C_5_H_14_ClNO, Sigma-Aldrich (St. Louis, MO, USA) 99%) (2:1 molar ratio) at a temperature of 70 °C for 1 h until a homogeneous colorless solution was obtained. Then, ZnSO_4_ was added to reline to obtain ChU0.3 and ChU0.1 DES electrolytes. A pre-drying process was performed to eliminate the crystallization water of choline chloride, urea and ZnSO_4_ powders by heating these powders separately at 120 °C under a rotary-pump vacuum for 2 days. After drying, the chemicals were transferred to an argon-filled glove box for the preparation of anhydrous reline, which was then used for the preparation of AChU. Ethaline was prepared with the same procedure as reline using ethylene glycol (C_2_H_6_O_2_) (reagent plus, ≥99% Sigma-Aldrich) instead of urea. ChU0.3 and ChEG were then selected as electrolytes for the mechanistic studies of Zn electrodeposition reported in Section 2.1 and Section 2.2.

### 3.2. Electrode Preparation and Cell Fabrication

**Figure 15:**
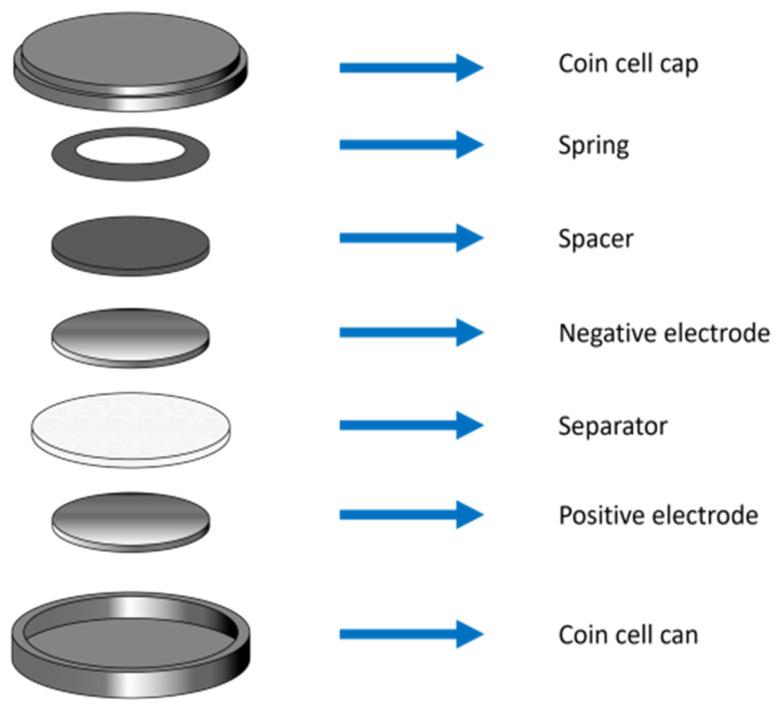
Schematic of the symmetric CR2032 coin cell and its components.

Cycling tests were carried out in symmetric CR2032 coin cells. The electrodes were Zn disks 12 mm in diameter, punched from 250 µm thick foil. The separator consisted of glass microfiber disks 19 mm in diameter and 260 µm thick. An amount of 350 μL of electrolyte, prepared as described in Section 3.1, was added to the cell. The layout of the CR2032 cells is shown in Figure 15.

### 3.3. Electrochemical Measurements

#### 3.3.1. Cyclic Voltammetry (CV)

CV measurements were performed in a three-electrode cell with an Ag/AgCl 3M KCl reference electrode (AMEL 373/SSG/12, Milano, Italy) and platinum counter electrode (CE). All potentials in this work are referenced to the Ag/AgCl 3 M KCl electrode. A glassy carbon (GC) electrode (AMEL 492/GC/3) with a surface area of 0.07 cm^2^, platinum (Pt) (AMEL 492/Pt/2) with a surface area of 0.03 cm^2^, gold (Au) (AMEL 492/Au/2) with a surface area of 0.03 cm^2^ and Zn foil with a surface area of 0.25 cm^2^ were employed as WEs. The cell contained 40 mL of electrolyte (see Section 3.1 for the composition), which was degassed by N2 bubbling (0.5 nlt min^−1^, 30 min) before running the experiment and kept under a blanket of flowing N_2_ during the electrochemical measurements. CVs were performed with a VMP-300 BioLogic potentiostat/galvanostat at 10 mV s^−1^. Specifically, before each experiment, the electrode was washed with deionized (DI) water, gently polished with alumina suspension 0.05 µm (ALLIED, Hanover, MD, USA) and finally rinsed with DI water and acetone. The RE and all cell components were thoroughly washed with DI water.

#### 3.3.2. Chronoamperometry (CA)

CA experiments were conducted using the same set-up mentioned above, using a Zn foil as the working electrode. A 3M ZnSO_4_ solution in ChU or ChEG was used at 25 °C imposing different potentials ranging from −1.25 to −1.8 V for 5 min.

#### 3.3.3. Galvanostatic Charge-Discharge Cycling (GCD)

GCD measurements were carried out with a NEWARE Battery Testing System (model: CT-4008T-5V10mA-164). The Zn∣Zn symmetric cells were cycled at 0.1, 0.2, 0.5 and 1 mA cm^−2^ for 30 min at each current density (CD), and the voltage was limited by setting the cut-off to ±2 V.

### 3.4. Scanning Electron Microscopy (SEM) Analysis

SEM images of deposited metallic Zn and Zn electrodes were acquired using a field emission-scanning electron microscope (FE-SEM, Zeiss SUPRA 40, Jena, Germany) operating in a high vacuum.

### 3.5. In Situ Surface-Enhanced Raman Spectroscopy

Raman spectra were recorded by means of a Jobin Yvon Horiba LabRam microprobe confocal system with the excitation at 633 nm provided by a He-Ne laser delivering 7 mW at the sample surface, with a 600 grid/mm spectrometer. A 50× long-working distance objective was used. In situ electrochemical measurements were performed in a Ventacon glass cell. The working electrode was a gold disc electrode, 0.6 mm in diameter, embedded in a Teflon holder; the counter electrode was a platinum wire loop concentric and coplanar with the working electrode, and the reference was an aqueous Ag/AgCl 3 M KCl electrode. In order to achieve SERS activity, the gold electrode was submitted to ORC treatment consisted of cycling the electrode in a separated cell in 0.1 M KCl solution, from 0.3 to 1.2 V at 500 mVs^−1^ fifty times. In situ Raman spectra were collected during potentiostatic polarization of the gold electrode at a series of potentials covering the span investigated by voltammetry.

## 4. Conclusions

In this work, two green, biocompatible and low-cost deep eutectic solvents (DESs) were studied as alternative electrolytes for rechargeable Zn batteries. We demonstrated that the addition of water could reduce the viscosity of the electrolyte to a level that ensures adequately low-anodic and -cathodic overpotentials and optimal discharge/charge cyclability. In addition, we compared the effects of the two hydrogen-bond donors (HBDs) considered, pointing out differences in cell life duration and failure mode. Specifically, cells with ChEG and ChU electrolytes fail by passivation and short circuits, respectively. Chronopotentiometric transients remain regular with the ChU0.3 electrolyte until cell failure, and this correlates well with multiple CV measurements showing the reversible plating/stripping of Zn in this system. It is worth noting that our cell cycling tests were carried out at higher current densities (CDs) and yielded longer lifetimes than in the literature. The unusual CVs patterns observed with gold and glassy carbon (GC) electrodes, featuring cathodic peaks on both the forward and backward peaks, were elucidated thanks to complementary electrochemical and in situ Raman measurements. In addition to the fundamental electrochemical insight gained in this work regarding Zn electrochemistry in DESs, we have highlighted that the ChU0.3 electrolyte is a promising candidate for rechargeable Zn batteries from the technological viewpoint, showing that implementation of hydrated DESs opens a new route for extended cyclability of rechargeable batteries with Zn anodes.

## Figures and Tables

**Table 1:** Recent studies on DESs as rechargeable Zn battery electrolytes.

DES Type	Cathode Type	Electrochemical Cell	Electrochemical Tests	Number of Cycles	Ref. Number
**ZnCl2:acetamide:H_2_O (1:3:1)**	Phenazine cathode	Symmetric and asymmetric coin cells	Symmetric: 0.1 and 0.05 mA h cm^−2^ Asymmetric: 0.1 mA cm^−2^ and 0.025 mA h cm^−2^	Over 10,000 cycles	[13]
**ZnCl_2_ in reline (anhydrous)**	δ-MnO_2_	Symmetric and asymmetric coin cells	Symmetric: ten cycles at 0.1, 0.2, 0.5 and 1.0 mA/cm2 for 30 min at each current density. Asymmetric: 50, 100, 150 and 200 mA/g of δ-MnO2 for ten cycles at each current density	150 cycles	[14]
**ZnCl2 in ethaline** **(anhydrous), ZnCl-EG**	NVO nanoribbon/CFP cathode,	Symmetric and asymmetric coin cells	Symmetric: ten cycles at different current densities of 0.1, 0.15, 0.2, 0.25 and 0.3 mA/cm^−2^ Asymmetric: 0.1 A g^−1^	200 cycles 300 cycles	[15]
**urea/LiTFSI/Zn(TFSI)2; TFSI, bis(trifluoromethanesulfonyl)imide)**	LiMn_2_O_4_ or LiFePO_4_	Coin cell or pouch cells	Cycling 0.05 mA/cm^2^ and 0.1 mA/cm^2^ with CV characterization	400 h 86.6% capacity retention after 600 cycles	[16]
**ZnCl_2_:acetamide**	Carbon electrode	Home-built cell for flow battery (hybrid RFBs)	Cycling 0.12 mA· cm^−2^	150 h	[17]
**Zn(ClO_4_)_2_·6H_2_O)** **and succinonitrile**	poly(2,3-dithiino-1,4-benzoquinone)	Coin cells	Cycling 0.3 C	3500 cycles	[32]
**sulfolane (SL) and Zn(ClO_4_)^2^·6H_2_O**	Polyaniline (PANI)	Symmetric and asymmetric coin cells	Cycling 0.5 mA/cm^2^	800 h 2500 times with a capacity of 72 mAh g^−1^ at 3 A g^−1^	[33]
**methylsulfonylmethane, zinc perchlorate**	NH_4_V_4_O_10_(NVO) or CaV_4_O_9_ (CVO)	Zn|Zn and asymmetric Zn|Cu coin cell or pouch cells	Cycling 0.05, 1 and 2 mA/cm^2^ with CV characterization	2000 h at 0.05 mA/cm^2^ 400 h at 2 mA/cm^2^ 76% retention after 3000 cycles at 3000 mA g^−1^	[34]

**Table 2:** Summary table of parameters that describe anodic–cathodic switching regime in galvanostatic cycling experiments with symmetric coin cells.

	ChU0.1	ChU0.3	AChU	ChEG
**D [cm^2^ s^−1^]**	3 × 10^−7^	3 × 10^−7^	3 × 10^−7^	3 × 10^−6^
**r_max_ [mm]**	0.05	0.05	0.05	0.037
**k_0,hs_ [cm s^−1^]**	3.5 × 10^−7^	3.5 × 10^−7^	3.5 × 10^−6^	3.5 × 10^−6^
**Shape**	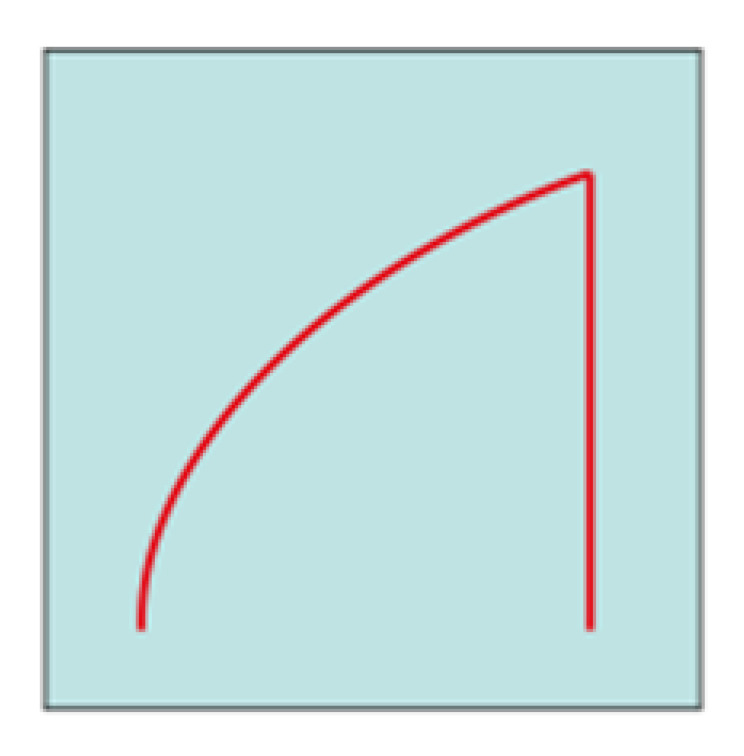	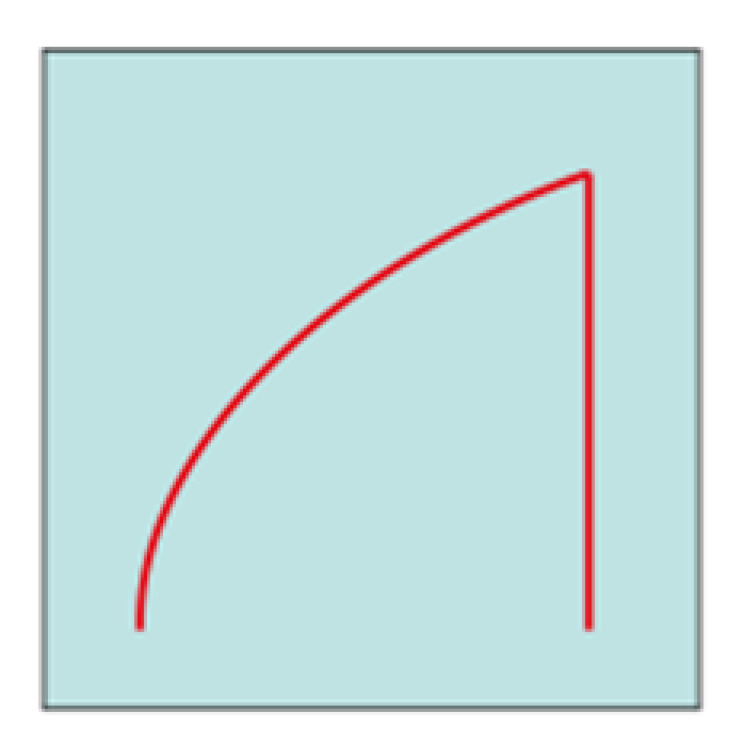	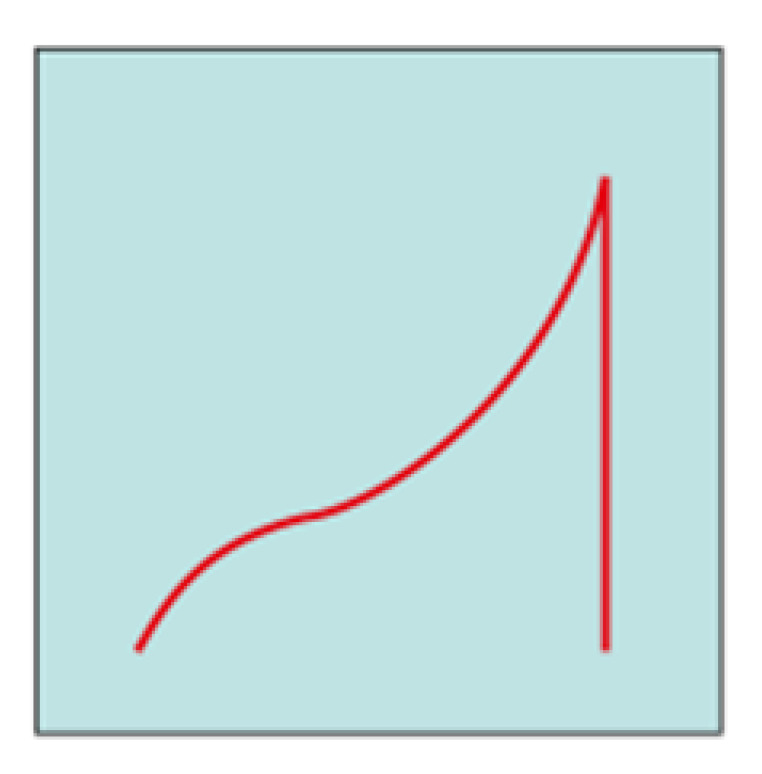	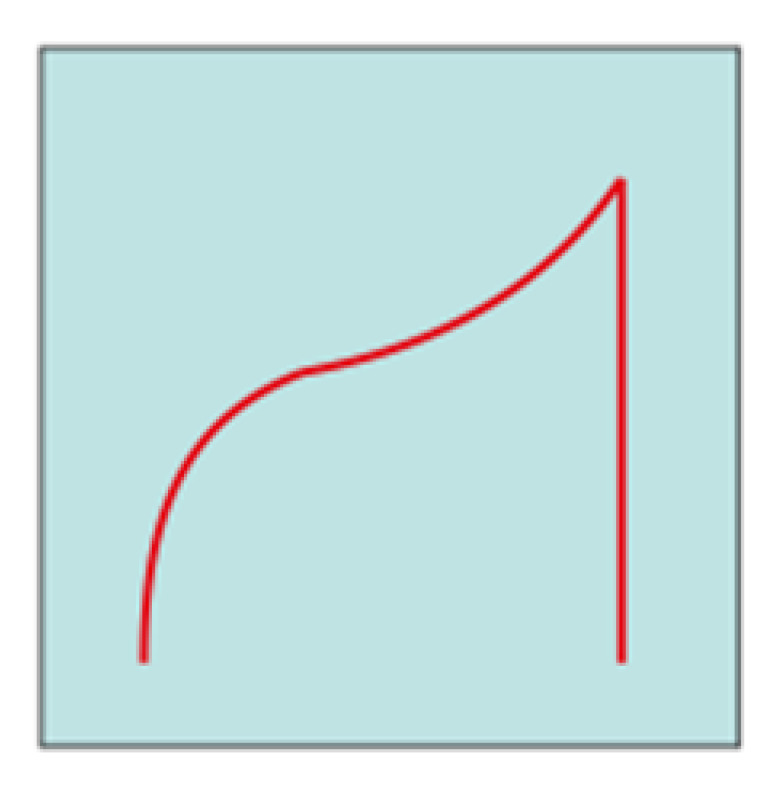

**Table 3:** F_C_ and k_pass_ values are estimated for the data reported in Figure 12 with the model of [49].

	ChU0.1	ChU0.3	AChU	ChEG
F_C_	1–450 cycles = 3.1 × 10^−7^ 450-end cycles = 0	1–400 cycles = 3.6 × 10^−7^ 400-end cycles = 0	1–30 cycles = 7 × 10^−6^ 30-end cycles = 0	0
k_pass_	0	0	4.14 × 10^−6^	4 × 10^−5^

## Data Availability

The data presented in this study are available on reasonable request from the corresponding author.
